# Development of Highly Active Bifunctional Electrocatalyst Using Co_3_O_4_ on Carbon Nanotubes for Oxygen Reduction and Oxygen Evolution

**DOI:** 10.1038/s41598-018-20974-1

**Published:** 2018-02-07

**Authors:** Mohammad Shamsuddin Ahmed, Byungchul Choi, Young-Bae Kim

**Affiliations:** Department of Mechanical Engineering, Chonnam National University, Gwangju, Republic of Korea

## Abstract

Replacement of precious platinum catalyst with efficient and cheap bifunctional alternatives would be significantly beneficial for electrocatalytic oxygen reduction reaction (ORR) and oxygen evolution reaction (OER) and the application of these catalysts in fuel cells is highly crucial. Despite numerous studies on electrocatalysts, the development of bifunctional electrocatalysts with comparatively better activity and low cost remains a big challenge. In this paper, we report a nanomaterial consisting of nanocactus-shaped Co_3_O_4_ grown on carbon nanotubes (Co_3_O_4_/CNTs) and employed as a bifunctional electrocatalyst for the simultaneous catalysis on ORR, and OER. The Co_3_O_4_/CNTs exhibit superior catalytic activity toward ORR and OER with the smallest potential difference (0.72 V) between the ${{\boldsymbol{E}}}_{{{\boldsymbol{j}}}_{{\bf{10}}}}$ (1.55 V) for OER and *E*_1/2_ (0.83 V) for ORR. Thus, Co_3_O_4_/CNTs are promising high-performance and cost-effective bifunctional catalysts for ORR and OER because of their overall superior catalytic activity and stability compared with 20 wt% Pt/C and RuO_2_, respectively. The superior catalytic activity arises from the unique nanocactus-like structure of Co_3_O_4_ and the synergetic effects of Co_3_O_4_ and CNTs.

## Introduction

Fabrication of hybrid nanomaterials that preserves improved properties other than the original properties of their base materials is an important issue in nanoscience and technology. Among the different allotropes of carbon nanomaterials, carbon nanotubes (CNTs) are one of the most promising nanomaterials for catalysis and sensing^1–4^. The catalytic sites in renewable and green energy systems need to be supported on conducting materials. These sites can be made of metallic nanostructures (such as nanoparticles and nanoflowers) or organometallic complexes. CNTs are potential ideal support material for electrocatalysts because of its electrical conductivity, high surface area, and relatively enhanced durability^5^. Noncovalent chemical approach to ensure a close incorporation between the CNTs and the metallic sites is promising because of its simple fabrication method and better preservation of the electronic properties of the CNTs without damaging the π-configuration.

Persistent environmental impact and increasing demands of traditional energy resources, such as, oil, gas, and coal have stimulated extensive efforts worldwide to develop renewable and green energy technologies, such as fuel cells (FCs) and water splitting systems^1,6–12^. Among many electrochemical reactions in FCs, the oxygen reduction reaction (ORR) is considered as the heart of FCs because it is the only reaction in cathode. By contrast, electrocatalytic oxygen evolution reaction (OER) through water splitting has been recognized as one of the most promising ways to generate oxygen^4^. Precious metals such as platinum (Pt) and/or its alloy materials have been most frequently used active electrocatalyst for both reactions^13–16^. However, Pt-based materials are susceptible to the poor durability and crossover effect in FCs^17,18^. Moreover, the high cost and bottleneck reserve in nature of Pt have also prohibited the full commercialization of FCs^19,20^. Meanwhile, transition metals and their alloys^21–24^ have been demonstrated as promising catalysts for ORR and/or OER. So far, the catalytic activities of many nonprecious metal electrocatalysts remain too low compared with those of noble metal catalysts. Moreover, the catalytic activity of the former electocatalysts is largely hindered by their inherent corrosion and oxidation susceptibility.

A number of cobalt oxides with uniform porous structure and large surface area have been employed as nonprecious catalysts to the electrochemical energy conversion reactions^25–29^. These catalysts have currently attracted much interest in the field of electrocatalysis because of their chemical and physical properties^29^. In particular, Co_3_O_4_ with spinel crystal structure is beneficial for the electron transport between Co^2+^ and Co^3+^ ions. Thus, Co_3_O_4_ has been widely considered as an efficient electrocatalyst for ORR and OER^30–33^. However, Co_3_O_4_ itself shows lower electrocatalytic activity because of its low electrical conductivity and dissolution, short active site density and agglomeration nature during electrocatalytic processes^30^. Nevertheless, further studies have exhibited that the synergy between carbon nanomaterials (i.e. graphene and CNTs) and Co_3_O_4_ can give a huge promotion of the electrocatalytic activity^26,28,34–37^. Many researches have investigated on Co_3_O_4_-based hybrid catalysts and obtained uniformly dispersed Co_3_O_4_ nanostructures (i.e. nanoparticles, core-shell, and hollow sphere) to improve electrocatalyst activity^30,38–41^. The results show that the size and shape of the nanocatalysts are deeply related to their electrocatalytic performances, and using these hybrid catalysts is considered one of the best strategies for improving catalytic activity and stability^42^.

**Figure 1:**
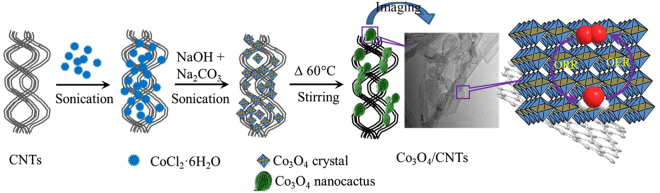
The schematic diagram of Co_3_O_4_/CNTs preparation and its catalytic activity.

Multifunctional (i.e. bifunctional or trifunctional)^28,41,43^ catalyst systems have been reported. However, a bifunctional catalyst system with enhanced activity for ORR and OER is difficult to develop because of the pH-dependent activity and stability^43^. OER electrocatalysts exhibit poor performance in alkaline electrolyte than acid electrolyte^44^. So far, doped-free and noncovalent CNTs with Co_3_O_4_-hybrid bifunctional catalyst for ORR, and OER has not been reported to date, although the N-doped graphene or CNTs with Co_3_O_4_-hybrid catalysts are rarely discussed^28,30^. Therefore, new strategies to develop a simply prepared, highly efficient, and cost-effective Co_3_O_4_- and CNT-based hybrid bifunctional electrocatalysts for ORR and OER is desirable for the large-scale production of clean energy. In this study, we have developed a simple strategy to synthesize Co_3_O_4_ nanocactus grown on CNTs (Co_3_O_4_/CNTs; in Fig. 1). For the first time, the Co_3_O_4_/CNTs were successfully employed as bifunctional Co_3_O_4_-based electrocatalyst for ORR and OER. The porous Co_3_O_4_/CNTs exhibited superior electrocatalytic activity and stability than the benchmarks Pt/C or RuO_2_ for ORR and OER, respectively, because of their high density of active sites and excellent charge transport capability.

## Experimental Section

The Co_3_O_4_/CNTs were synthesized by a simple chemical method. In a typical preparation, CoCl_2_.6H_2_O (62 mg) was mixed with 30 mL of water. Then, 30 mg of multiwalled CNTs, which were treated with acid for 30 min^45^, were mixed with 30 mL of water. The solution was added into the CoCl_2_.6H_2_O solution and vigorously stirred for 1 h. The pH was controlled by slowly adding a solution of 0.21 mol L^−1^ NaOH and 0.066 mol L^−1^ Na_2_CO_3_ under vigorous stirring until pH 10 was reached at room temperature (RT). The prepared suspension was kept at 60 °C for next 24 h under gentle stirring. Finally, the Co_3_O_4_/CNTs were filtered and washed with water for and dried at 60 °C. The bare CNTs were prepared using the same protocol but without addition of CoCl_2_.6H_2_O solution. Also, the Co_3_O_4_/CNTs in various pH values (i.e. pH 7, 12, 14) were prepared by controlled addition of aforementioned alkaline solution. The electrochemical and instrumental characterizations were described in the supporting information.

## Results and Discussions

Figure 1 shows that the Co_3_O_4_ precursor and clean CNTs were mixed with a simple cooperative assembly of prepared alkaline solution in water at RT. The solution was then kept at 60 °C under gentle stirring for next 24 h to form Co_3_O_4_ nanocactus onto CNTs. The growth of Co_3_O_4_ nanocactus probably goes through a modified mechanism as below^46^-

1 $${\rm{NaOH}}+{{\rm{Na}}}_{2}{{\rm{CO}}}_{3}\to 3{{\rm{Na}}}^{+}+{{\rm{OH}}}^{-}+{{\rm{CO}}}_{3}^{2-}$$

2 $${{\rm{CoCl}}}_{2}\to {{\rm{Co}}}^{2+}+2{{\rm{Cl}}}^{\mbox{--}}$$

3 $${{\rm{Co}}}^{2+}+2{{\rm{Cl}}}^{\mbox{--}}+2{{\rm{Na}}}^{+}+2{{\rm{OH}}}^{\mbox{--}}\to {\rm{Co}}{({\rm{OH}})}_{2}+2{\rm{NaCl}}$$

4 $$4{\rm{Co}}{({\rm{OH}})}_{2}+2{{\rm{CO}}}_{3}^{2-}+{{\rm{OH}}}^{-}\to 4{\rm{CoOOH}}+2{{\rm{CO}}}_{2}\uparrow +2{{\rm{H}}}_{2}{\rm{O}}$$

5 $$2{\rm{CoOOH}}+{\rm{Co}}{({\rm{OH}})}_{2}\to {{\rm{Co}}}_{3}{{\rm{O}}}_{4}\downarrow +2{{\rm{H}}}_{2}{\rm{O}}$$

**Figure 2:**
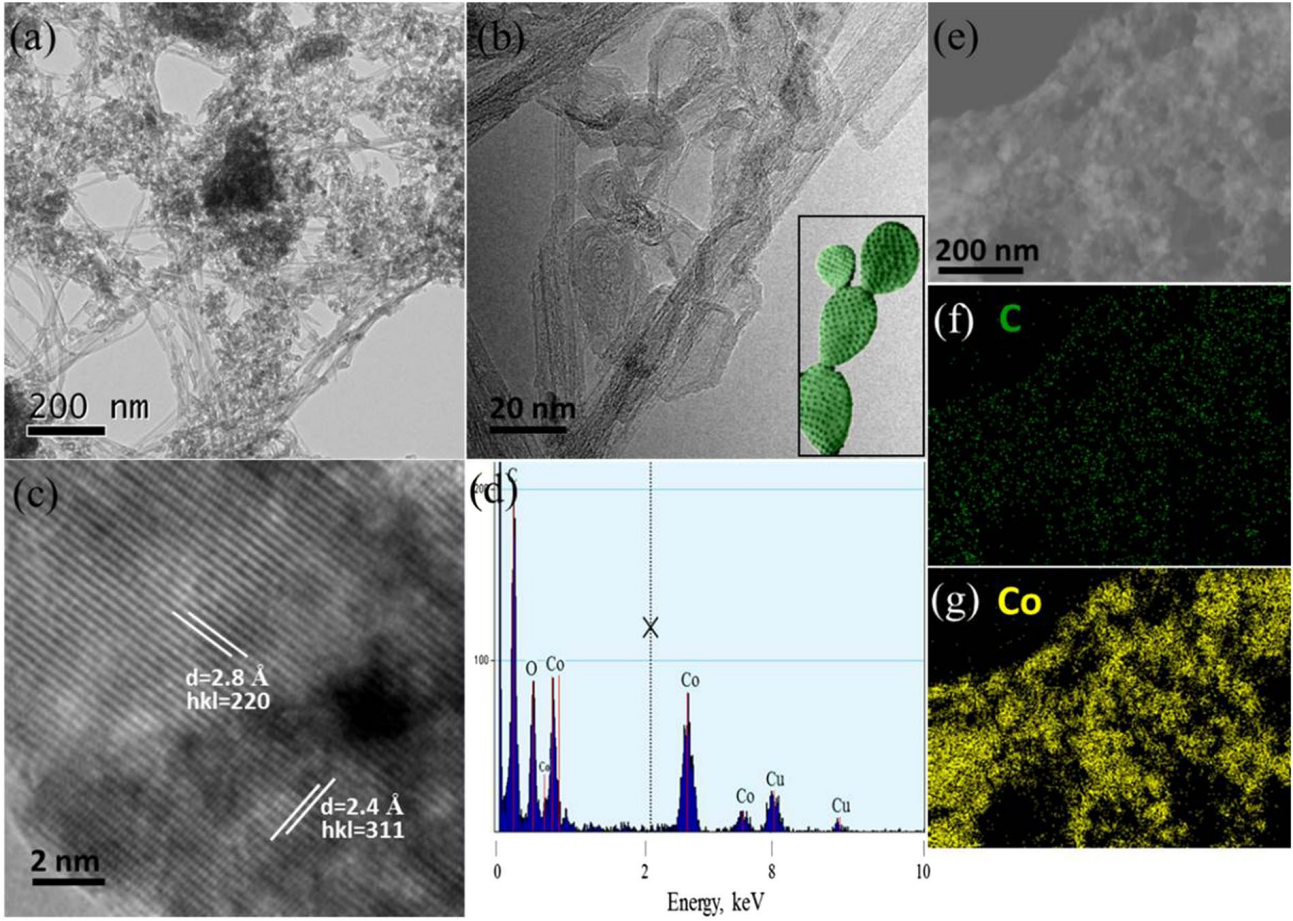
TEM (**a** and **b**), HRTEM (**c**) images, EDX spectrum (**d**), bright-fiend TEM image (**e**) and C (**f**), Co (**g**) elemental mapping of Co_3_O_4_/CNTs, insets: a photograph of a microdasys cactus, enlarged HRTEM images.

However, the as prepared Co_3_O_4_/CNTs were found with a highly crystalline form. The growth of Co_3_O_4_ nanocactus grown on CNTs (Figure S1) was confirmed by transmission electron microscopy (TEM) analysis in Fig. 2. TEM revealed that the numerous nano-sized Co_3_O_4_ cactus were grown onto CNTs (Fig. 2a) and the average size of a single unit of nanocactus was ~25 nm in length with ~5 nm thick sidewall (Fig. 2b). High resolution TEM (HRTEM) showed the crystalline spinel structure of the Co_3_O_4_ nanocactus (Fig. 2c) and the lattice spacing of 2.4 Å and 2.8 Å can be assigned to the (311) and (220) planes of typical Co_3_O_4_^47^. The bulk elemental component of Co_3_O_4_/CNTs was investigated by energy dispersive X-ray spectroscopy (EDX) in Fig. 2d. The C peak at 0.2 keV was accompanied by an O peak in the EDX spectra. Three Co peaks at ~0.77, 6.9 and 7.63 keV corresponding to CoL_α1_, CoL_β1_ and CoL_γ1_, respectively, were also obtained in the EDX spectra. The as-prepared Co_3_O_4_/CNTs consisted of 6.93 wt% Co, 81.93 wt% C, and 11.14 wt% O. Also, Fig. 2 shows bright-fiend TEM image (e) and C (f), Co (g) elemental mapping of Co_3_O_4_/CNTs sample which confirming once again the presence of C and Co elements.

**Figure 3:**
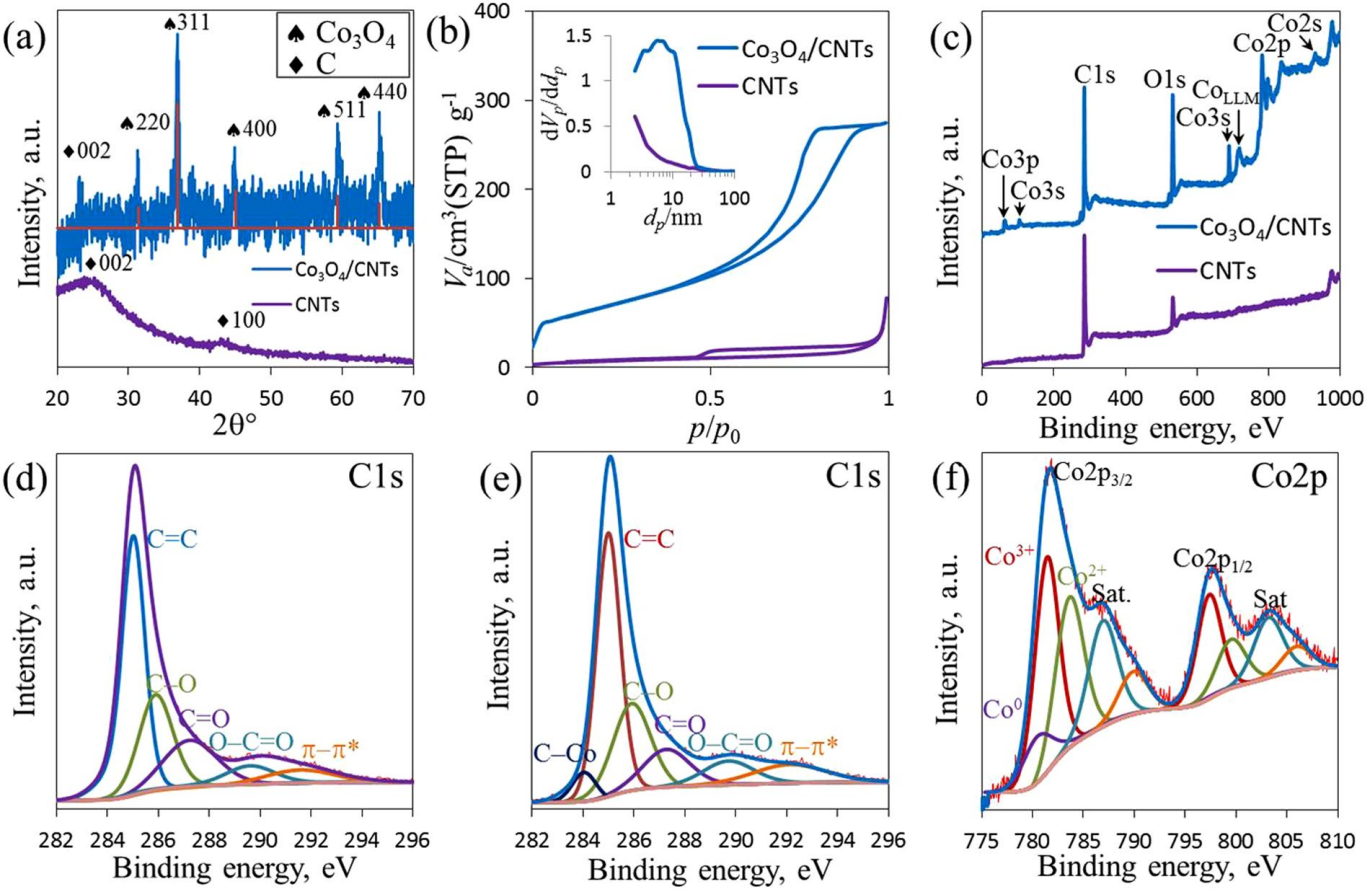
The XRD spectra (**a**), the nitrogen adsorption–desorption isotherms (**b**), XPS spectra (**c**), core level of C1s (**d** and **e**), and Co2p XPS spectrum (**f**) of CNTs and Co_3_O_4_/CNTs; inset: the corresponding pore-size distribution (**b**).

The TEM results are consistent with the X-ray diffraction (XRD) data. XRD was performed to investigate the phase structure of Co_3_O_4_/CNTs. In Fig. 3a, several peaks of the pristine Co_3_O_4_ were consistent with the standard Co_3_O_4_ (ICDD: 98-008-8940, red line). Except for the broad peak (002) at ~25°, which may be ascribed to disordered stacked graphitic structure of CNTs, the major diffraction peaks of Co_3_O_4_/CNTs were in good agreement with those of Co_3_O_4_^48–50^. The type IV N_2_ adsorption/desorption isotherm curve with a distinct hysteresis loop in the relative pressure range of 0.45–0.99 confirmed the presence of mesopores in Co_3_O_4_/CNTs and bare CNTs samples (Fig. 3b). The Brunaue–Emmett–Teller specific surface area (SSA) for Co_3_O_4_/CNTs was measured to be 373 m^2^ g^−1^, which was approximately 3-magnitudes higher than the corresponding typical values for Co_3_O_4_-decorated carbon nanomaterials (i.e., Co_3_O_4_/N-rGO, 103.9 m^2^ g^−1^; Co@Co_3_O_4_/NC–1, 111 m^2^ g^−1^; Co_3_O_4_/CNW-A, 166 m^2^ g^−1^)^28,30,32^. On the contrary, the SSA for bare CNTs was 133.2 m^2^ g^−1^. Barrett–Joyner–Halenda pore size distribution curves confirm the presence of the main mesopores with various sizes between 3 nm and 25 nm (average pore diameter, 6.9 nm) and a pore volume of 1.32 cm^3^ g^−1^. The average pore diameter and pore volume were much higher than those of bare CNTs (Fig. 3b inset). Therefore, a large SSA, high pore volume, and wide pore size distribution are the clear indication of facile electrocatalysis on Co_3_O_4_/CNTs sample.

X-ray photoelectron spectroscopy (XPS) was performed to elucidate the chemical changes and confirmed the cobalt state during Co_3_O_4_ growth on CNTs. The peaks obtained in the XPS spectra at 284.2, 531.0 and 780.7 eV (Fig. 3c) could be ascribed to C1s, O1s and Co2p, respectively, due to the existence of carbon, oxygen and cobalt in Co_3_O_4_/CNTs. Significant difference was observed in the presence of Co_3_O_4_ in Co_3_O_4_/CNTs compared with bare CNTs. The high-resolution C1s XPS spectra of bare CNTs (Fig. 3d) and Co_3_O_4_/CNTs (Fig. 3e) represent the defective sp^3^-carbon and basal-plane sp^2^-carbon of CNTs^51^. Both figures showed four absorbance peaks for oxygenated sp^3^-carbon at 285.8, 287.3, and 289.7 eV, which were attributed to C–O, C=O, and O–C=O, respectively, including distinct oxygen-free sp^2^-carbon (C=C) at 285.0 eV^52^. Moreover, a tiny shakeup peak was obtained at 292.2 eV for π–π*, signifying higher degree of graphitization^53,54^. The tiny peak at low binding energy of 284.1 eV could probably be attributed to the C–Co bond in Co_3_O_4_/CNTs^55^. The overall elemental composition of Co_3_O_4_/CNTs is listed in Table S1. Furthermore, XPS confirmed the oxidized state of the Co-species with the detection of binding energies of 781.7 eV and 797.6 eV which were attributed to Co2p_3/2_ and Co2p_1/2_ peaks, respectively (Fig. 3f)^56,57^. However, the Co^0^, Co^3+^ and Co^2+^ species were detected at 781.4, 781.6, and 783.8 eV in Co2p_3/2_ with corresponding satellite peak (786.7 eV) due to the presence of Co_3_O_4_ in the Co_3_O_4_/CNTs sample. At Co2p_1/2_, the Co^3+^ and Co^2+^ species also appeared at 797.45 eV and 799.6 eV with its shakeup satellite at 803.3 eV. Moreover, the numerical analysis of XPS data was also recorded, and Co was detected as 6.94 wt% with a good ratio of Co^3+^/Co^2+^ (1.1) at pH 10 which was the lowest value among all pHs (Figure S2 and Table S2).

### Electrochemical ORR on Co_3_O_4_/CNTs

**Figure 4:**
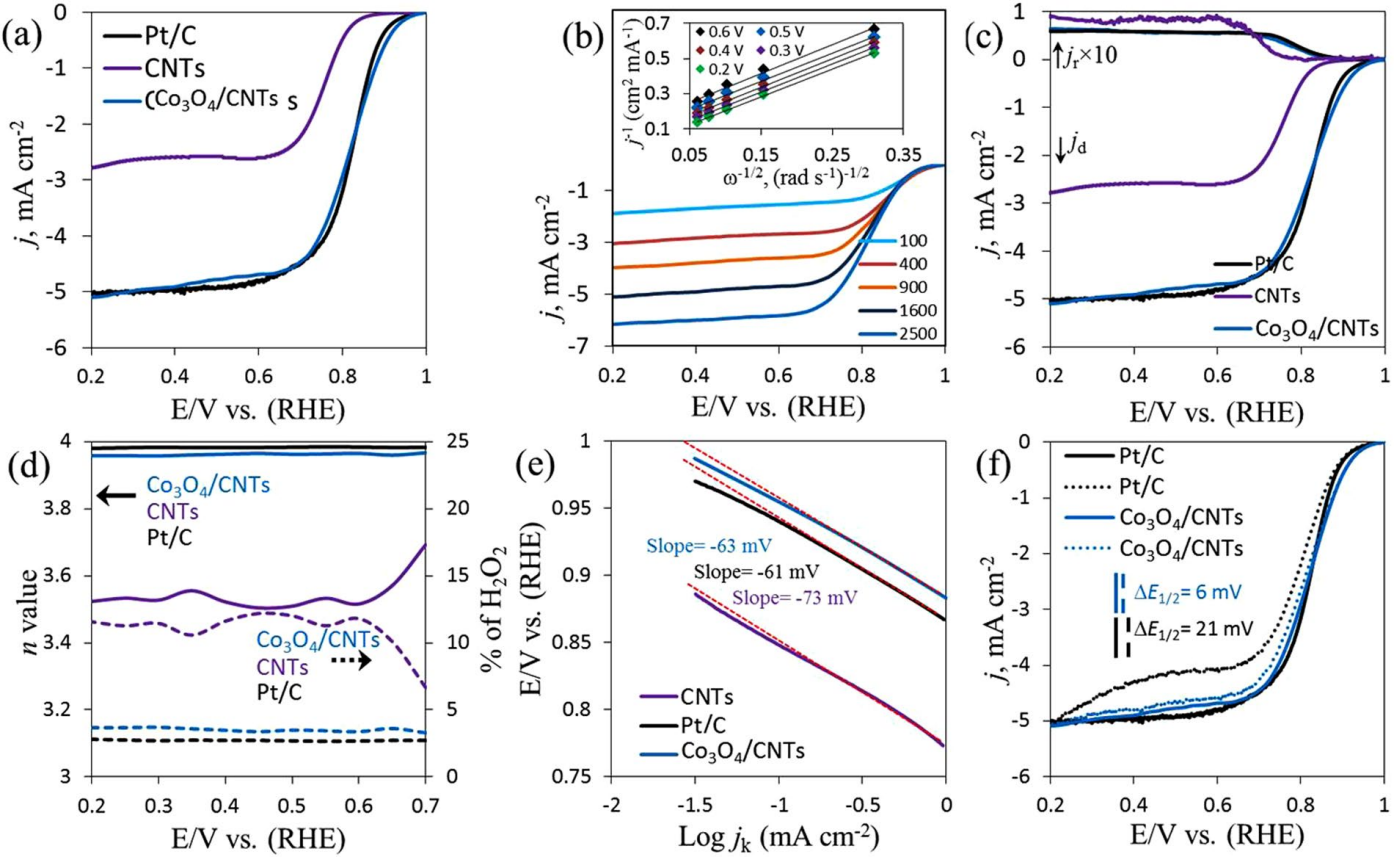
LSV curves for ORR on CNTs, Co_3_O_4_/CNTs and Pt/C catalyst in O_2_-saturated 0.1 M KOH solution at a scan rate of 5 mV s^−1^ and at a rotating speed of 1600 rpm (**a**), LSV curves on Co_3_O_4_/CNTs in same electrolyte at various rotating speeds and K–L plots in inset (**b**), RRDE curves for ORR at 1600 rpm with a constant applied potential of 0.8 V vs. RHE on the ring electrode (**c**), the transferred electron number and the corresponding H_2_O_2_ synthesis during ORR (**d**), Tafel plots (**e**) on CNTs, Co_3_O_4_/CNTs and Pt/C electrodes, and LSV curves on Co_3_O_4_/CNTs and Pt/C before (solid lines) and after (dotted lines) 3000 cycles (**f**).

The linear sweep voltammogram (LSV) curves on rotating disk electrode (RDE) exhibited ORR for Co_3_O_4_/CNTs, bare CNTs (catalyst mass loading, 153 μg cm^−2^) and 20 wt% Pt/C electrodes in O_2_-saturated 0.1 M KOH solution at a scan rate of 5 mV s^−1^ and at 1600 rpm (Fig. 4a) and signify the electrocatalytic ORR performance on all electrodes. The superior electrocatalytic ORR was observed on Co_3_O_4_/CNTs in terms of the improved onset potential (*E*_onset_) of 0.93 V and a half-wave potential (*E*_1/2_) of 0.83 V than the CNTs (*E*_onset_, 0.83 V and *E*_1/2_, 0.76 V (Figure S3). These values were also superior to those of commercially available Pt/C (*E*_onset_ of 0.91 V and *E*_1/2_ of 0.83 V) and several other reported Co_3_O_4_-based catalysts for ORR^26,58^. Moreover, the current density (*j*, normalized by electrode area, 0.196 cm^2^) at the Co_3_O_4_/CNTs electrode was higher than that of the CNTs modified electrode and closer to that of Pt/C. Thus, the Co_3_O_4_/CNTs showed better electrocatalytic activity for ORR in terms of *E*_onset_, *E*_1/2_, and *j*. This result highlights the importance of the incorporation of nanocactus-shaped Co_3_O_4_ with CNTs that have mesoporous structure and higher SSA. The ORR dynamics at the Co_3_O_4_/CNTs electrode were then investigated by RDE, and the results are shown in Fig. 4b. Figure 4b displays a series of RDE curves for ORR using the Co_3_O_4_/CNTs catalyst at various rotation speeds in same electrolyte at 5 mV s^−1^ scan rate. The obtained data were analyzed using Koutecky–Levich (K−L) equation as follows^58–60^:

6 $$\frac{1}{j}=\frac{1}{{j}_{k}}+\frac{1}{{j}_{L}}$$

7 $$jL=B{\omega }^{1/2}=0.62nFA{D}_{{O}_{2}}^{2/3}{C}_{{O}_{2}}{v}^{-1/6}{\omega }^{1/2}$$

8 $${j}_{k}=nFk{{C}}_{{O}_{2}}$$

where *j*, *j*_k_, and *j*_L_ are the measured, kinetic, and diffusion limiting current densities (mA cm^−2^), respectively; *n* is the electron transfer number per O_2_, and *A* is the surface area of the working electrode, Moreover, *F* and *T* are Faraday constant (96485.3 C mol^−1^) and temperature, respectively; ${D}_{{O}_{2}}$ and ${C}_{{O}_{2}}$ are the oxygen diffusion coefficient (1.9 × 10^−5^ cm^2^ s^−1^) and the bulk concentration (1.2 mM L^−1^), respectively, in 0.1 M KOH^60^; *v* is the kinetic viscosity of the electrolyte (1 × 10^−2^ cm^2^ s^−1^); *ω* is the angular velocity of electrode (2π*rpm), and *k* is the electron-transfer rate constant. Based on the K–L equation, a plot of *j*_k_^−1^ vs. *ω*^−1/2^ was yield a straight line and the slopes of those plots reflect the *B* factor in equation (7).

However, Fig. 4b inset shows the K–L plots for Co_3_O_4_/CNTs electrode and the slopes of all K–L plots remain approximately constant over the studied potential range. This result indicates the number of electrons transferred in the ORR remained constant. Based on equations (6) and (7), the average *n* value in ORR was estimated to be 4, suggesting a four-electron (4*e*^−^) pathway for electrocatalytic ORR^61–63^. The ORR dynamics on the CNTs electrode were also investigated by RDE and the average *n* value in ORR was estimated to be 3.6 from corresponding K–L plots (Figure S4). The *j*_k_ obtained from the intercept of the K–L plots for the Co_3_O_4_/CNTs (16.5 mA cm^−2^ at 0.8 V) was 3.1-magnitudes larger than that of bare CNTs (5.2 mA cm^−2^) catalyst and similar to that of Pt/C (17.3 mA cm^−2^). The ORR activities on as-synthesized Co_3_O_4_/CNTs at various pH values were also investigated (Figure S5a). Although the Co_3_O_4_/CNTs @ pH 12 has the highest Co_3_O_4_ (Table S2), the relatively low Co^3+^/Co^2+^ might lead to a high charge-transfer (Figure S5b). Hence, a relatively better electrocatalytic activity was observed at Co_3_O_4_/CNTs @ pH 10 in based on the higher *j*_k_ among all pH-dependent Co_3_O_4_/CNTs (Figure S5a inset).

The rotating ring–disk electrode (RRDE) measurement was performed to further evaluate the ORR pathway on Co_3_O_4_/CNTs, bare CNTs, and Pt/C electrodes. The Co_3_O_4_/CNTs electrode exhibited high disk current density (*j*_d_) for ORR and much lower ring current density (*j*_r_) than CNTs. The *j*_r_ profiles accompanied with further reduction of peroxide species synthesized during ORR process are shown in the upper curves. Both *j*_d_ and *j*_r_ from Pt/C are very similar to that of Co_3_O_4_/CNTs.

The RRDE data were used to further verify the transferred electron number and monitor the corresponding H_2_O_2_ formation on aforementioned three electrodes during ORR process from equations (9) and (10)^28,64^ in Fig. 4d. The average *n* value for ORR at the Co_3_O_4_/CNTs electrode (3.96) was consistently higher than that at the CNTs (3.6) over the tested potential range of 0.7–0.2 V (vs. RHE). The corresponding H_2_O_2_ yields were 3.5% and 9.6% for Co_3_O_4_/CNTs and CNTs, respectively, over the same potential range. The calculated *n* value (3.98) and H_2_O_2_ (2.9%) yield on Pt/C were slightly higher than the Co_3_O_4_/CNTs. The calculated *n* values are similar to the result obtained from the K–L plots, signifying that the ORR on Co_3_O_4_/CNTs hybrid was mainly by 4*e*^−^ involved pathway and the main byproduct was H_2_O.

9 $$n=\frac{4{i}_{d}}{{i}_{d}+\frac{{i}_{r}}{N}}$$

10 $${H}_{2}{O}_{2} \% =\frac{200\frac{{i}_{r}}{N}}{{i}_{d}+\frac{{i}_{r}}{N}}$$

11 $$N=\frac{-{i}_{r}}{{i}_{d}}$$

where *N* is the collection efficiency of the RRDE (0.37), and *i*_d_ and *i*_r_ are the disk and ring electrode currents, respectively.

The estimated *j*_k_ values were plotted against the electrode potential to investigate the Tafel behavior of Co_3_O_4_/CNTs, CNTs and Pt/C (Fig. 4e). The better ORR activity on Co_3_O_4_/CNTs was further confirmed by the lower Tafel slope of 63 mV dec^−1^ at low overpotential (*η*) than the CNTs (73 mV dec^−1^) and similar to that of Pt/C (61 mV dec^−1^). Furthermore, the LSV curves before and after the accelerated degradation test (ADT) on Co_3_O_4_/CNTs and Pt/C in Fig. 4f. It was found that the *E*_1/2_ shifted largely at the negative direction (21 mV), and *j*_L_ lost 17.9% on Pt/C after 3000 consecutive cycles. By contrast, the 3.5- and 9-magnitudes lower *E*_1/2_ shift (6 mV) and *j*_L_ loss (2%) were observed on the Co_3_O_4_/CNTs under the same conditions. Moreover, Figure S6a shows the current density as the function of time by chronoamperometry technique for Co_3_O_4_/CNTs and Pt/C at an applied potential of 0.8 V. The *j* was maintained up to 93% after 20 h run in real condition, indicating that the catalytic activity on Co_3_O_4_/CNTs could be sustained for a long time. For Pt/C, the catalytic activity was then maintained up to 76% with the same period of time. In addition, the Co_3_O_4_/CNTs electrode demonstrated good methanol tolerance than the Pt/C (Figure S6b). The TEM image of used Co_3_O_4_/CNTs displays the decay morphology with the crystalline nature of Co_3_O_4_ after 20 h of real-time continuous monitoring. These results indicate that the Co_3_O_4_/CNTs are a competent ORR electrocatalyst because of its better electrocatalytic activity, fuel selectivity, and operational stability than the Pt/C.

### Electrochemical OER on Co_3_O_4_/CNTs

**Figure 5:**
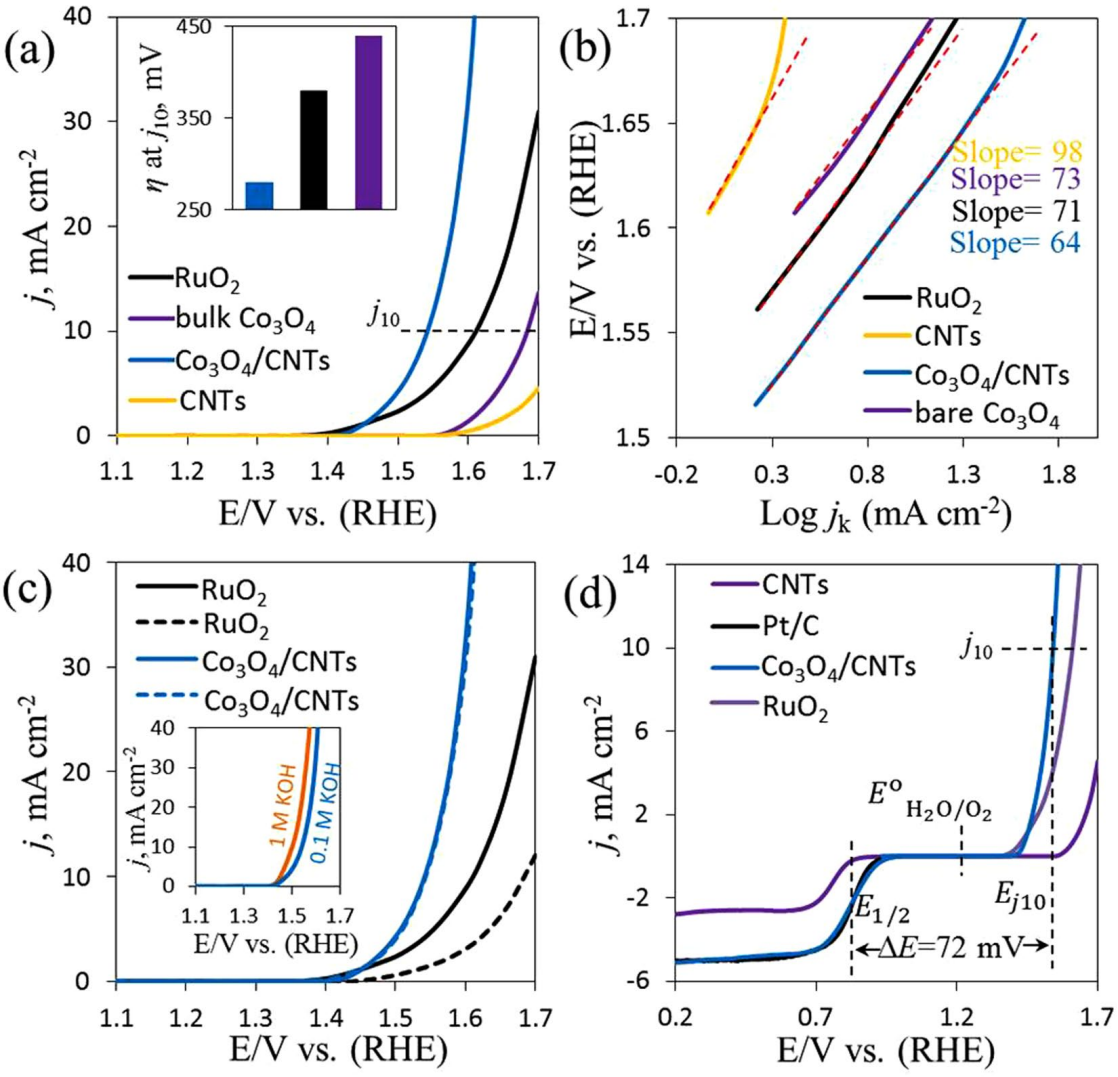
Comparison of the OER activity of CNTs, bulk Co_3_O_4_, Co_3_O_4_/CNTs and RuO_2_ electrodes by LSV (**a**), corresponding Tafel plots of those electrodes (**b**), LSV curves for the 1^st^ and 1000^th^ potential cycles (**c**), bifunctional catalytic activities of CNTs, Co_3_O_4_/CNTs and benchmarks Pt/C or RuO_2_ catalysts toward both ORR and OER (**d**); the overall LSV curves in the potential range of 0.2 to 1.7 V was investigated in argon-saturated 0.1 M KOH solution at 5 mV s^−1^ scan rate and at the rotating speed of 1600 rpm. Insets: the comparison of overpotential at *j*_10_ (**a**) and OER activity of Co_3_O_4_/CNTs in argon-saturated 0.1 and 1 M KOH solution (**c**).

To evaluate the potential use of our hybrid catalyst, we employed Co_3_O_4_/CNTs electrode to evaluate the electrocatalytic OER. The OER catalytic activities of all catalysts were studied by LSV at 5 mV s^−1^. The Co_3_O_4_/CNTs were used with same mass loading and afforded higher OER activity than either bare bulk Co_3_O_4_, CNTs or RuO_2_ in Fig. 5. Figure 5a shows that the *E*_onset_ for bulk Co_3_O_4_ was 1.55 V and the maximum *j* was 13.6 mA cm^−2^ at 1.7 V. The *E*_onset_ for RuO_2_ was 1.36 V with maximum of *j* = 31 mA cm^−2^ at the same electrode potential. However, considerable negative shifted in the *E*_onset_ was observed at Co_3_O_4_/CNTs (1.43 V) with highest *j* = 70.8 mA cm^−2^ at 1.7 V and the CNTs showed the lowest performance than all electrodes. However, the Co_3_O_4_/CNTs electrode showed a potential of 1.55 V at the current density of 10 mA cm^−2^ $({E}_{{j}_{10}})$, which was lower than that of bulk Co_3_O_4_ (1.68 V) and RuO_2_ (1.61 V). Moreover, as shown in Fig. 5a inset, the *η* required to drive a *j*_10_ for the Co_3_O_4_/CNTs was 280 mV, which was also significantly lower than that for the bulk Co_3_O_4_ and RuO_2_ (440 mV and 380 mV, respectively). Thus, the Co_3_O_4_/CNTs exhibited higher OER activity than the bulk Co_3_O_4_, CNTs and RuO_2_ electrodes in respect to the *η* and *j*. This result indicating that the Co_3_O_4_/CNTs have higher density of active sites than bulk Co_3_O_4_ and CNTs, and the porous Co_3_O_4_ served as the better active catalytic site for the superior OER even better than benchmark RuO_2_ which resulted in the synergic effect of Co_3_O_4_ and CNTs, the porous nanocactus-like structure, and better Co^3+^/Co^2+^ ratio. These characteristics allowed improved ability for electron transfer. The poor current densities of bare CNTs and Co_3_O_4_ were probably due to the degradation nature of carbon^32^ and aggregation with less conducive nature, respectively^26^.

The Tafel slope of each catalyst beyond the *E*_onset_ was calculated to understand in detail the OER mechanism and the result are shown Fig. 5b. Tafel plots display a lower Tafel slope of 64 mV dec^−1^ for Co_3_O_4_/CNTs than those of RuO_2_ (71 mV dec^−1^), bulk Co_3_O_4_ (73 mV dec^−1^) and CNTs (98 mV dec^−1^), indicating more favorable kinetics toward OER on the Co_3_O_4_/CNTs electrode^32,65^. The Tafel slope of Co_3_O_4_/CNTs was also comparable to other reported Co_3_O_4_-based OER catalysts^26,66^. The OER mechanism can be assumed as follows according to the Tafel slope of Co_3_O_4_/CNTs. The OER on active Co−O-system^65^ in Co_3_O_4_/CNTs catalyst was initiated by water adsorption and the formation of adsorbed (ads) reactive intermediate, ${{\rm{OH}}}_{{\rm{abs}}}^{\ast }$, by releasing a proton and electron in equation (12). Afterwards, this ${{\rm{OH}}}_{{\rm{abs}}}^{\ast }$ was converted to another type OH, ${{\rm{OH}}}_{{\rm{abs}}}^{\ast }$, in equation (13) (both OH are chemically same but energetically different). In equation (14), a second proton and electron transfer yielded an oxide intermediate, and this step is a rate-determining step (RDS). Recombination of two oxide intermediates completed one reaction turnover in equation (15)^67^.

12 $$\mathrm{Co}\mbox{--}{\rm{O}}+{{\rm{H}}}_{2}{\rm{O}}\to {{\rm{O}}\mbox{--}\mathrm{Co}\mbox{--}\mathrm{OH}}_{{\rm{abs}}}^{\ast }+{{\rm{H}}}^{+}{+{\rm{e}}}^{-}$$

13 $${{\rm{O}}\mbox{--}\mathrm{Co}\mbox{--}\mathrm{OH}}_{{\rm{abs}}}^{\ast }\to {\rm{O}}\mbox{--}\mathrm{Co}-{{\rm{OH}}}_{{\rm{abs}}}$$

14 $${\rm{O}}\mbox{--}\mathrm{Co}\mbox{--}{{\rm{OH}}}_{{\rm{abs}}}\,\mathop{\longrightarrow }\limits^{RDS}\,{{\rm{O}}\mbox{--}\mathrm{Co}\mbox{--}{\rm{O}}}_{{\rm{abs}}}+{{\rm{H}}}^{+}+{{\rm{e}}}^{-}$$

15 $${{\rm{O}}\mbox{--}\mathrm{Co}\mbox{--}{\rm{O}}}_{{\rm{abs}}}+{{\rm{O}}\mbox{--}\mathrm{Co}\mbox{--}{\rm{O}}}_{{\rm{abs}}}\to 2\mathrm{Co}\mbox{--}{\rm{O}}+{{\rm{O}}}_{2}\uparrow $$

Furthermore, the electrochemical stability of Co_3_O_4_/CNTs electrode was also compared with RuO_2_ under a fixed *η* and electrolyte conditions. Good stability of Co_3_O_4_/CNTs electrode was confirmed by the similar LSV curves measured at the 1^st^ and 1000^th^ potential cycles (Fig. 5c). The used catalyst was characterized by XPS, and the results suggest that amperometric operation did not change significantly in the chemical states except the increase in C–O bond in CNTs and satellite band in cobalt (Figure S7). These results also suggest that Co_3_O_4_/CNTs have longer stability in electrochemical process than the RuO_2_. The OER processes at Co_3_O_4_/CNTs electrode in 0.1 and 1 M KOH exhibit the same *E*_onset_ of 1.43 V (Fig. 5c inset). At higher potentials, the more rapid increase in current density was observed for 1 M KOH owing to higher conductivity of the electrolyte. At 270 mV overpotential, *j* = 22 mA cm^−2^ was obtained in 1 M KOH solution, while it was 10 mA cm^−2^ in 0.1 M KOH.

The overall oxygen activity of the Co_3_O_4_/CNTs as a bifunctional catalyst could be evaluated (Fig. 5d) by the potential difference (Δ*E*) between the ${E}_{{j}_{10}}$ for OER and *E*_1/2_ for ORR^68^. However, the Co_3_O_4_/CNTs catalyst showed the smallest Δ*E* of 0.72 V and this value was markedly lower than the Δ*E* obtained using commercial Pt/C (0.85 V) and many other Co- and Co_3_O_4_-based materials i.e., Co-N/G-600, 0.96 V; Co@Co_3_O_4_/NC-1, 0.85 V; Co_3_O_4_/N-Gas, 0.79 V^21,30,67^. This result signifies better reversible oxygen electrode. The detailed comparison with various Co- and Co_3_O_4_-based materials is shown in Table 1. These results clearly indicate that the Co_3_O_4_/CNTs catalyst is a promising low-cost and efficient catalyst for both ORR and OER.

**Table 1:** The bifunctional catalytic activity of Co_3_O_4_/CNTs catalyst for ORR and OER.

Name	Electrolyte	*E*_1/2_ (V vs. RHE)	${{\bf{E}}}_{{{\boldsymbol{j}}}_{{\boldsymbol{10}}}}$ (V vs. RHE)	Δ*E* (V)	References
Co_3_O_4_/CNTs	0.1 M KOH	0.83	1.55	0.72	This work
Co-N/G-600	0.1 M KOH	0.764*	1.724*	0.96	^21^
Co_3_O_4_/N-rmGO	0.1 M KOH	0.83	1.54	0.71	^26^
Co_3_O_4_/NPC	0.1 M KOH	0.74*	1.63*	0.89	^28^
Co@Co_3_O_4_/NC-1	0.1 M KOH	0.80	1.65	0.85	^30^
Co_3_O_4_/CNW-B	0.1 M KOH	0.755*	1.556*	0.801	^37^
Co_3_O_4_/N-GAs	0.1 M KOH	0.87	1.66	0.79	^66^
CMO/N-rGO	0.1 M KOH	0.80	1.66	0.86	^69^
Co_9_S_8_(600)/N,S-GO	0.1 M KOH	0.75	1.63	0.88	^70^
Co_x_O_y_/NC	0.1 M KOH	0.80	1.66	0.86	^71^

*Converted V vs. RHE.

## Conclusion

We demonstrated an easy and generic method to synthesize a unique nanocactus-like structure of Co_3_O_4_ material embedded onto CNTs for bifunctional electrocatalysis. The newly developed Co_3_O_4_/CNTs were an effective bifunctional ORR and OER electrocatalyst with comparatively better activities and stability than the Pt/C or RuO_2_ because of their unique architecture with large surface area, rich active sites, and good electron transfer properties. The excellent catalysis and stability of Co_3_O_4_/CNTs with abundant active sites could be attributed to the strong interaction between the nanocactus-shaped Co_3_O_4_ and CNTs. Thus, Co_3_O_4_/CNTs are promising alternatives to noble metal-based catalysts for FCs and water splitting applications because of their low-cost, facile synthesis, and excellent catalysis and stability.

## Electronic supplementary material

Dataset 1
