# Supplementary material for: Development of Highly Active Bifunctional Electrocatalyst Using Co3O4 on Carbon Nanotubes for Oxygen Reduction and Oxygen Evolution
Source: Sci Rep. 2018 Feb 7;8:2543. doi: 10.1038/s41598-018-20974-1 (PMC5803219; doi:10.1038/s41598-018-20974-1)
Supplement: Supplementary file 1 — Dataset 1 [file 41598_2018_20974_MOESM1_ESM.doc]

Supporting Information of:

Co_3_O_4_ Nanocactus on Carbon Nanotubes: High-Performance Bifunctional Electroactive Catalyst for Oxygen Reduction and Oxygen Evolution

Mohammad Shamsuddin Ahmed, Byungchul Choi, Young-Bae Kim*

Department of Mechanical Engineering, Chonnam National University, Gwangju, Republic of Korea. Tel.: +82 62 5301677, E-mail: [suddin_bcsir@yahoo.com](mailto:suddin_bcsir@yahoo.com) (MS Ahmed), [bcchoi@jnu.ac.kr](mailto:bcchoi@jnu.ac.kr) (B Choi), [ybkim@chonnam.ac.kr](mailto:ybkim@chonnam.ac.kr) (YB Kim)*

*Electrochemical Performance*

All electrochemical experiments including cyclic voltammetric (CV) and chronoamperometric (CA) techniques were performed using a three-electrode potentiostat (CHI 700C electrochemical workstation, CH instrument, USA). A standard three-electrode setup was used with a working electrode of glassy carbon (GC)-RRDE (disk outer diameter, ring inner diameter, ring outer diameter are 5 mm, 6.5 mm and 7.5 mm, respectively), a Pt wire and an Ag/AgCl (3M KCl) were used as counter electrode and reference electrode, respectively. All potentials were calculated with respect to reversible hydrogen electrode (RHE) scale according to the Nernst equation (*E*_RHE_ = *E*_Ag/AgCl_ + 0.059pH + *E*°_0.197 V_, at 25 °C). The electrolyte was used O_2_ or argon (Ar)-saturated 0.1 M KOH aqueous solution at room temperature (RT, ~25 °C). A 3 mg mL^−1^ suspension in water was prepared by introducing a predetermined amount of Co_3_O_4_/CNTs under sonication. Then 10 µL of the prepared catalyst ink (153 μg cm^−2^) was dropped onto the surface of prepolished with 0.05 µM alumina slurry GC-RRDE and allowed to dry in air at RT. The commercially available Pt/C (Johnson Matthey 20 wt% on Vulcan XC-72) and RuO_2_ suspension was prepared by dispersing 1 mg mL^−1^ of Pt/C or RuO_2_ in ethanol containing 5 μL of 5% Nafion solution (in alcohol). Afterwards, 10 μL of Pt/C or RuO_2_ was loaded onto the GC-RRDE.

*Instrumental Characterization*

The transmission electron microscopy (TEM) and energy dispersive X-ray spectroscopy (EDX) were carried out using a Tecnai 20 microscope at 200 kV, Crystal structure was examined by X-ray diffraction (XRD), which was carried out on a Rigaku D/max-2500, using filtered Cu Kα radiation. Detailed chemical compositions of the samples were analyzed by X-ray photoelectron spectroscopy (XPS) using a VG multilab 2000 spectrometer (Thermo VG Scientific, Southend-on-Sea, Essex, UK) in an ultrahigh vacuum using an unmonochromatized Mg Kα (1253.6 eV) radiation source and a spherical section analyzer. BET surface area and pore size distribution were obtained through the Barrett-Joyner-Halenda method by nitrogen isotherm adsorption and desorption (BelsorpII mini, BEL Japan Inc.).


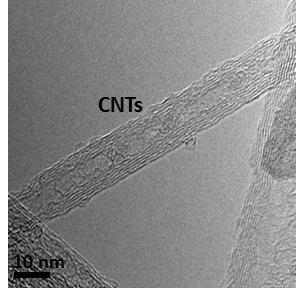


**Figure S1:** TEM image of CNTs.


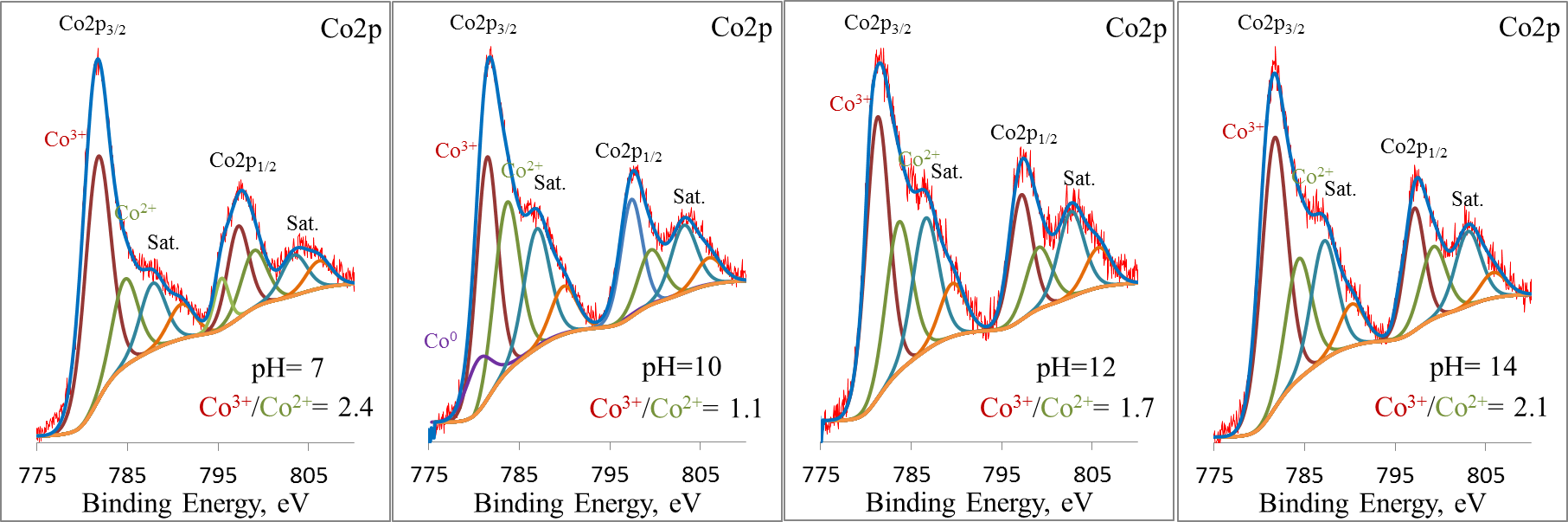


**Figure S2:** The pH dependent (pH 7-14) core level of Co2p XPS spectra of Co_3_O_4_/CNTs.

**Table S1:** The numerical analysis of XPS data (as wt%) for bare CNTs and Co_3_O_4_/CNTs.

|  | CNTs | Co_3_O_4_/CNTs |
| --- | --- | --- |
| C1s | 95.57 | 81.91 |
| O1s | 4.43 | 11.16 |
| Co2p |  | 6.94 |

**Table S2:** The numerical analysis of XPS data for Co_3_O_4_/CNTs prepared in different pH values.

| Co2p wt% | pH 7 | pH 10 | pH 12 | pH 14 |
| --- | --- | --- | --- | --- |
| Co^3+^ | 3.6 | 3.3 | 4.4 | 4.1 |
| Co^2+^ | 1.5 | 2.8 | 2.6 | 2.0 |
| Co^0^ | 0.5 | 0.8 | 0.1 | 0.6 |
| Total | 5.57 | 6.93 | 7.10 | 6.70 |
| Ratio | 2.4 | 1.1 | 1.7 | 2.1 |


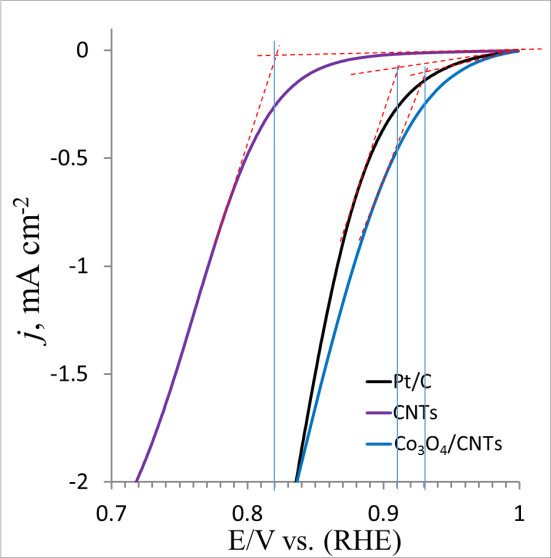


**Figure S3:** Enlarged LSV curves of ORR on CNTs Co_3_O_4_/CNTs and P/C in O_2_-saturated 0.1 M KOH at a scan rate of 10 mV s^–1^ for identifying *E*_onset_.


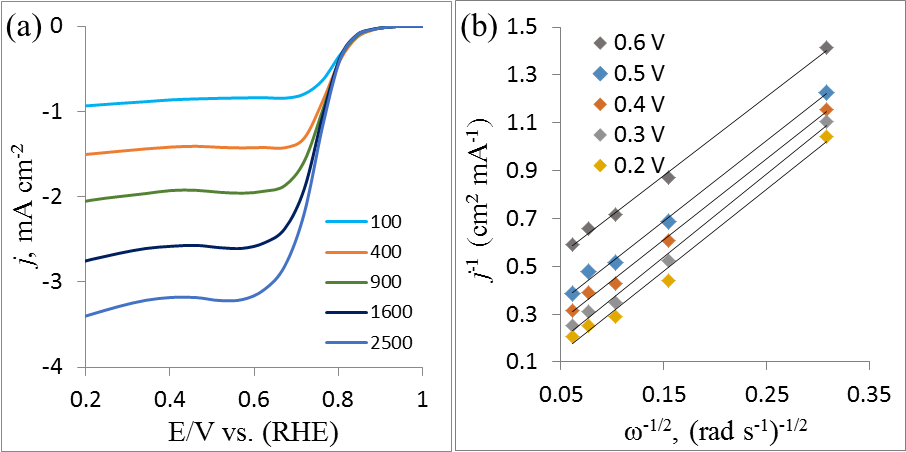


**Figure S4:** LSV curves of ORR on CNTs in O_2_-saturated 0.1 M KOH at various rotating speeds and at a scan rate of 10 mV s^–1^ (a) and K–L plots (b).


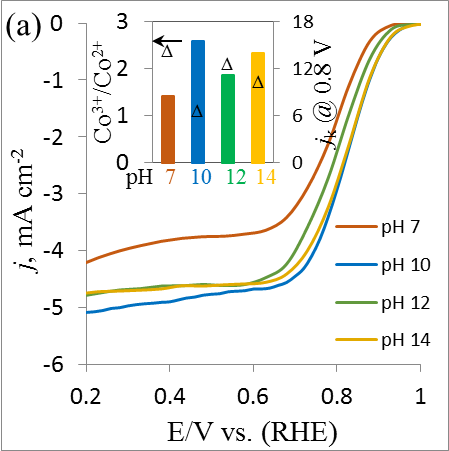

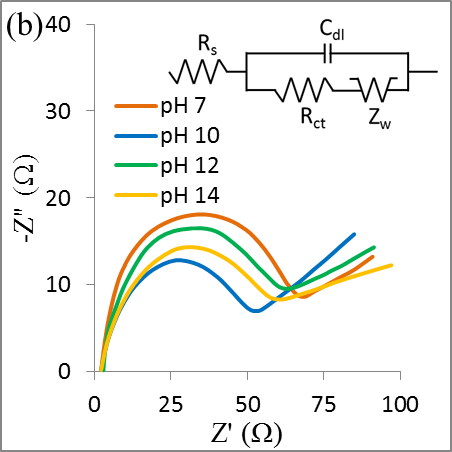


**Figure S5:** LSV curves of ORR on all as prepared Co_3_O_4_/CNTs @ various pH values in O_2_-saturated 0.1 M KOH at 1600 rpm and at a scan rate of 10 mV s^–1^ (a) and the electrochemical impedance spectra of all prepared Co_3_O_4_/CNTs in 0.1 M KOH (b); inset: the corresponding calculated *J*_k_ at 0.8 V (vs. RHE) with Co^3+^/Co^2+^ (a) and the corresponding circuit diagram (b).


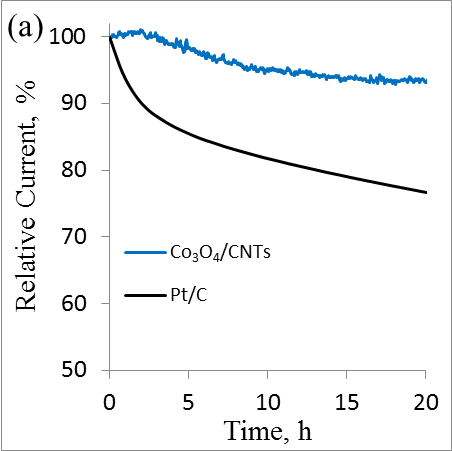

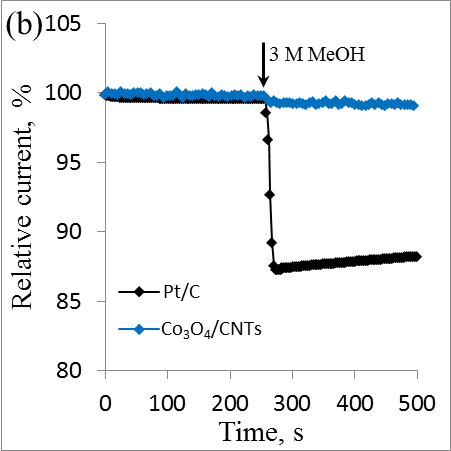


**Figure S6:** The amperometric response for stability (a) and selectivity (b) tests for ORR of Co_3_O_4_/CNTs and Pt/C in O_2_-saturated 0.1 M KOH at an applied potential of 0.8 V (vs. RHE), arrow indicates the addition of 3 M methanol into the electrochemical cell.


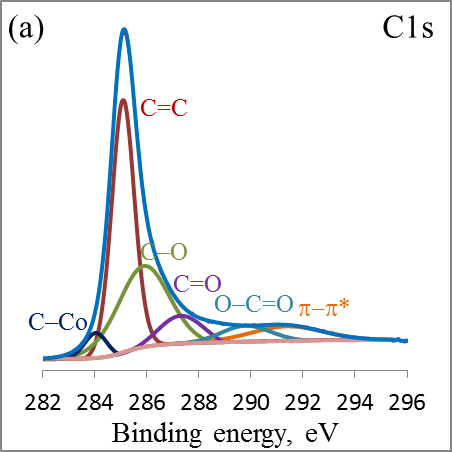

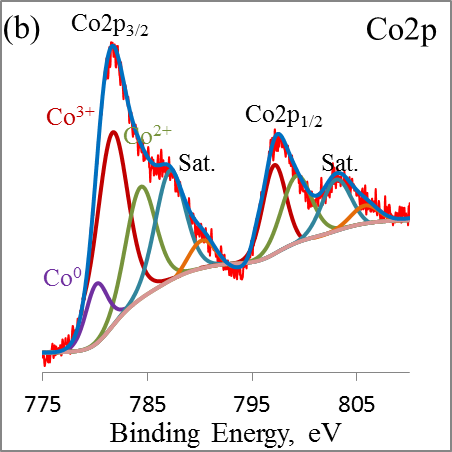


**Figure S7:** The core level C1s (a) and Co2p (b) XPS spectra of Co_3_O_4_/CNTs after 20 h amperometric stability test.
